# Plasma inflammatory and immune proteins as predictors of intra-amniotic infection and spontaneous preterm delivery in women with preterm labor: a retrospective study

**DOI:** 10.1186/s12884-018-1780-7

**Published:** 2018-05-09

**Authors:** Hyunsoo Park, Kyo Hoon Park, Yu Mi Kim, Song Yi Kook, Se Jeong Jeon, Ha-Na Yoo

**Affiliations:** 1 Department of Obstetrics and Gynecology, Seoul National University College of Medicine, Seoul National University Bundang Hospital, 82, Gumi-ro 173 Beon-gil, Seongnamsi, Kyeonggido 463-707 Korea; 2 Center for High-risk Pregnancy and Neonate, Seoul National University Bundang Hospital, Seongnam, Korea

**Keywords:** Interleukin-6, Intra-amniotic infection, Maternal plasma, Preterm labor, Proteins, Spontaneous preterm delivery

## Abstract

**Background:**

We investigated whether various inflammatory and immune proteins in plasma predict intra-amniotic infection and imminent preterm delivery in women with preterm labor and compared their predictive ability with that of amniotic fluid (AF) interleukin (IL)-6 and serum C-reactive protein (CRP).

**Methods:**

This retrospective cohort study included 173 consecutive women with preterm labor who underwent amniocentesis for diagnosis of infection and/or inflammation in the AF. The AF was cultured, and assayed for IL-6. CRP levels and cervical length by transvaginal ultrasound were measured at the time of amniocentesis. The stored maternal plasma was assayed for IL-6, matrix metalloproteinase (MMP)-9, and complements C3a and C5a using ELISA kits. The primary and secondary outcome criteria were positive AF cultures and spontaneous preterm delivery (SPTD) within 48 h, respectively. Univariate, multivariate, and receiver operating characteristic analysis were used for the statistical analysis.

**Results:**

In bivariate analyses, elevated plasma IL-6 level was significantly associated with intra-amniotic infection and imminent preterm delivery, whereas elevated plasma levels of MMP-9, C3a, and C5a were not associated with these two outcomes. On multivariate analyses, an elevated plasma IL-6 level was significantly associated with intra-amniotic infection and imminent preterm delivery after adjusting for confounders, including high serum CRP levels and short cervical length. In predicting intra-amniotic infection, the area under the curve (AUC) was significantly lower for plasma IL-6 than for AF IL-6 but was similar to that for serum CRP. Differences in the AUCs between plasma IL-6, AF IL-6, and serum CRP were not statistically significant in predicting imminent preterm delivery.

**Conclusions:**

Maternal plasma IL-6 independently predicts intra-amniotic infection in women with preterm labor; however, it has worse diagnostic performance than that of AF IL-6 and similar performance to that of serum CRP. To predict imminent preterm delivery, plasma IL-6 had an overall diagnostic performance similar to that of AF IL-6 and serum CRP. Plasma MMP-9, C3a, and C5a levels could not predict intra-amniotic infection or imminent preterm delivery.

## Background

Preterm labor and intact membranes are responsible for approximately one-third of preterm births, and are associated with significant perinatal mortality and morbidity [1, 2]. Adverse neonatal outcomes are closely related to the occurrence of complications caused by the degree of prematurity and intra-uterine infection [3–5]. Therefore, given a reported intra-amniotic infection rate of 10% [2, 6], a more accurate prediction of intra-amniotic infection and preterm delivery, especially using noninvasive methods, is clinically important for improving pregnancy management and for counseling women with preterm labor.

Traditionally, measuring inflammatory and immune proteins in amniotic fluid (AF) is thought to be of value in predicting intra-amniotic infection and preterm birth in women with preterm labor. Several studies have shown that increased levels of interleukin (IL)-6, matrix metalloproteinase (MMP)-9, and complement split products (C3a and C4a) in AF are associated with both intra-amniotic infection and preterm parturition [7–11]. However, their clinical use is currently limited by the requirement of invasive AF sampling. In this regard, noninvasive approaches that analyze these proteins in a maternal blood sample may provide a safe alternative to traditional invasive amniocentesis because intra-uterine infection and preterm parturition may lead to a significant increase in several cytokines simultaneously in both the AF and maternal blood compartments [12–16]. A few studies have reported the role of these inflammatory and immune proteins in a maternal blood sample, particularly in preterm labor. Some of these studies are limited by a small sample size [17], a cross-sectional study design (which could not establish a well-established association, unlike a longitudinal design) [18, 19], and no adjustment for confounding factors [13, 19]. This study aimed to determine whether the various inflammatory and immune proteins in plasma are predictive of intra-amniotic infection and imminent preterm delivery (defined as spontaneous preterm delivery [SPTD] within 48 h) in women with preterm labor and to compare their predictive ability with that of AF IL-6 and serum C-reactive protein (CRP).

## Methods

### Study population

A retrospective cohort study was conducted on consecutive singleton pregnant women diagnosed with preterm labor (23 + 0 to 33 + 6 weeks of gestation) who were admitted to Seoul National University Bundang Hospital (Seongnamsi, Republic of Korea) from June 2004 to April 2015. The inclusion criteria were: (1) a live fetus; (2) an aliquot of maternal plasma available for analysis; (3) amniocentesis performed to evaluate the infectious and inflammatory status of AF at the time of enrollment; and (4) cervical length measured at the time of amniocentesis. The exclusion criteria were: (1) multifetal pregnancy; (2) preterm premature rupture of the membranes (pPROM); (3) prior or subsequent cerclage; (4) active labor at enrollment (defined as the presence of cervical dilatation greater than 3 cm); (5) evidence of clinical chorioamnionitis; and (6) major fetal congenital anomalies. Preterm labor was defined as the presence of regular uterine contractions, with a frequency of at least 2 contractions every 10 min, and cervical change (softening, effacement, or dilation) that required hospitalization. Gestational age was determined based on the last menstrual period and the first or second trimester (≤20 weeks) ultrasound results, when available. The ethics committee at Seoul National University Bundang Hospital approved the study (IRB no. B-1105/128–102). During the study period, amniocentesis for retrieval of AF and maternal blood sampling were immediately offered to patients who were admitted to our institution. Patients provided written informed consent for the collection and use of the blood and AF samples and for the use of their clinical information for research purposes. The primary and secondary outcome measures were positive AF cultures and SPTD within 48 h, respectively.

### Amniotic fluid, sample collection and preparation

After obtaining informed consent, a transabdominal amniocentesis was performed under sonographic guidance with a 22-gauge spinal needle under aseptic conditions, and 15 to 20 mL of AF was aspirated. The AF was immediately sent to the laboratory for culture of aerobic/anaerobic bacteria and genital mycoplasmas (*Ureaplasma urealyticum* and *Mycoplasma hominis*) using previously described methods [20]. The remaining AF was centrifuged at 1500 *g* and 4 °C for 10 min, aliquoted, and stored at − 70 °C for further analysis. The IL-6 levels in the stored AF were measured using the enzyme-linked immunosorbent assay (ELISA) human DuoSet Kit (R&D Systems, Minneapolis, MN, USA). The range of the IL-6 standard curve was 7.8–600 pg/mL. The intra- and inter-assay coefficients of variation were < 10% each. CRP and cervical length by transvaginal ultrasound were measured in participants at the time of amniocentesis as part of the hospital protocol using methods that have been previously described [20, 21]. The results of AF culture, CRP, and cervical length measurement were available to the managing clinicians, but the AF IL-6 results were not.

### Inflammatory and immune mediator assays in plasma

At the time of amniocentesis, maternal blood samples were obtained from all participants and were collected in EDTA tubes. The samples were centrifuged at 1500 *g* at 4 °C for 10 min, and the supernatant was aliquoted and stored at − 70 °C until assayed. The stored plasma samples were assayed for multiple inflammatory and immune proteins (IL-6, MMP-9, C3a and C5a). ELISA kits were used to measure IL-6 (R&D System, Minneapolis, MN, USA), MMP-9 (DuoSet ELISA from R&D System, Minneapolis, MN, USA) and complement C3a and C5a (BD Biosciences, San Diego, CA 92121, USA) levels in the plasma samples, according to the manufacturer’s instructions. The ranges of the IL-6, MMP-9, C3a, and C5a standard curves were 0.2–10 pg/mL, 31.2–2000 pg/mL, 0.078–2.5 ng/mL, and 0.08–2.5 ng/mL, respectively. The intra- and inter-assay coefficients of variation were 3.6 and 10.9% for IL-6, 2.9 and 18.3% for MMP-9, 10.4 and 13.7% for C3a, and 7.9 and 8.0% for C5a, respectively.

### Management of preterm labor and definitions of various factors

Intra-amniotic infection was defined as the presence of a positive AF culture for microorganisms. The patients were hydrated and, if uterine contractions persisted, they were started on a regimen of intravenous tocolysis with magnesium sulfate, ritodrine, or atosiban. Corticosteroids were administered between 24 and 34 weeks of gestation. The decision to use prophylactic antibiotics was left to the discretion of the attending obstetrician. At our institution, antibiotic treatment of women with preterm labor for pregnancy prolongation is not recommended except for group B streptococcus prophylaxis or treatment of cases with clinically suspected (AF WBC count ≥ 50 cells/mm^3^) or diagnosed intra-amniotic infection, as previously described in detail [20]. Although the regimen for antibiotic prophylaxis in these patients varied according to the study period and the obstetrician, ampicillin and azithromycin (clarithromycin or erythromycin) were the main antibiotics used. For women with intra-amniotic infection, antibiotics are selected on the basis of the type of microorganisms found in the AF cultures. Medications such as antibiotics, corticosteroids, and tocolytics were started after sampling. A histologic diagnosis of chorioamnionitis was made in accordance with the definition previously described in detail [22]. Clinical chorioamnionitis was diagnosed in accordance with the criteria proposed by Gibbs et al. [23].

### Statistical methods

Data were analyzed using SPSS version 22.0 for Windows (IBM Corp., Armonk, NY, USA). The Shapiro–Wilk test was used to determine whether the data in groups were distributed normally. Univariate analysis was performed using the Student’s *t*-test or Mann–Whitney *U* test for continuous data and the *χ*^2^-test or Fisher’s exact test for dichotomous data. A multivariate logistic regression model was used to examine the relationship of the level of each protein in plasma to the outcome after adjusting for baseline variables, with a *P* value < 0.1 in univariate analysis. Independent variables associated with invasive amniocentesis were not included in the logistic regression model, because these were not baseline variables but were used for comparison. In the logistic regression model, continuous data were transformed into dichotomous data to decrease the problem of multicollinearity, especially between the serum CRP and plasma IL-6 (*r* = 0.495) or for prediction or decision-making purposes. Receiver-operating characteristic (ROC) curves were used to identify the best cut-off values for dichotomization. The optimal cut-off values were selected on the basis of the Youden index (maximum [sensitivity + specificity] − 1). The areas under the ROC curves (AUCs) for each protein and the cervical length were measured, and compared using the method described by DeLong et al. [24]. The sensitivity, specificity, and predictive values of plasma IL-6 and cervical length with reference to AF IL-6 and serum CRP levels were calculated and compared using the McNemar test. The sampling-to-delivery interval was assessed using Kaplan–Meier analysis and was compared between groups using the log-rank test. The Cox proportional model was used to determine whether higher levels of various proteins in plasma were associated with the sampling-to-delivery interval after adjusting for other prognostic variables. The Spearman rank correlation test was used to measure the relationship between the continuous variables that did not follow a normal distribution. All *P* values were two-sided, and *P* < .05 was considered statistically significant.

## Results

In total, 173 women with a diagnosis of preterm labor who met all the inclusion criteria were finally included. Their mean gestational ages (± standard deviation) at sampling and at delivery were 30.1 ± 2.7 weeks and 35.3 ± 3.9 weeks, respectively. The prevalence of intra-amniotic infection was 9.2% (16/173), and the microbes isolated from the AF in 16 patients were *Ureaplasma urealyticum* (*n* = 16) and *Mycoplasma hominis* (*n* = 13). Polymicrobial invasion was present in 13 of the 16 patients (81.2%). SPTD within 48 h of sampling occurred in 15.0% (26/173) of the patients.

Of the measured plasma proteins (IL-6, MMP-9, C3a, and C5a), positive significant correlations were found only between C5a and C3a (*r* = 0.376, *P* < 0.001) and between C5a and MMP-9 (*r* = 0.166, *P* = 0.029), and only the plasma IL-6 level was significantly correlated with serum CRP level (*r* = 0.494, *P* < 0.001) and AF IL-6 level (*r* = 0.289, *P* < 0.001). Cervical length was significantly correlated with the plasma MMP-9 level (*r* = − 0.269, *P* < 0.001) and serum CRP (*r* = − 0.159, *P* = 0.036), while other immune-related proteins measured in plasma were not correlated with cervical length (all variables, *r* = − 0.114 to − 0.040, *P* > 0.05).

The demographic and clinical characteristics of the study population according to AF culture results are presented in Table 1. Table 2 describes the clinical characteristics of the study population according to the occurrence of SPTD within 48 h after sampling.

**Table 1:** Characteristics of the study population according to amniotic fluid culture results

	Amniotic fluid culture		*P-*value
	Positive (*n* = 16)	Negative (*n* = 157)	
Maternal age (years)	31.3 ± 4.9	31.6 ± 4.0	0.638
Nulliparity	56% (9)	62% (97)	0.666
Gestational age at sampling (weeks)	28.3 ± 3.0	30.2 ± 2.6	0.014
Gestational age at delivery (weeks)	30.0 ± 3.1	35.9 ± 3.5	< 0.001
Serum C-reactive protein (mg/L)	25.3 ± 21.5	7.5 ± 11.6	< 0.001
Cervical length on ultrasonography (mm)	19.2 ± 8.9	25.3 ± 12.1	0.054
Plasma C3a (μg/mL)	13.8 ± 6.8	12.8 ± 7.9	0.366
Plasma C5a (ng/mL)	46.0 ± 10.3	44.8 ± 13.0	0.712
Plasma interleukin-6 (pg/mL)	9.9 ± 9.3	4.6 ± 6.1	0.001
Plasma matrix metalloproteinase-9 (ng/ml)	248.7 ± 232.0	210.6 ± 185.0	0.405
Use of tocolytic agents	94% (15)	94% (147)	0.985
Use of antibiotics	75% (12)	26% (40)	< 0.001
Use of antenatal corticosteroids	100% (17)	75% (120)	0.026
Clinical chorioamnionitis	6% (1)	0% (0)	0.002
Histological chorioamnionitis^a^	75% (12/16)	32% (27/85)	0.001
Amniotic fluid interleukin-6 (ng/mL)	26.3 ± 21.4	1.9 ± 7.7	< 0.001

Data are given as mean ± standard deviation or % (n/N)

^a^Data for the histologic evaluation of the placenta were only available for 101 of the 173 women because delivery took place at another institution in 5 cases, and histologic evaluation of the placenta was not performed in 67 cases because of our institutional policy that only the placentas from preterm deliveries are sent for histopathologic examination

**Table 2:** Characteristics of the study population according to the occurrence of delivery within 48 h

	Spontaneous preterm delivery after sampling		*P-*value
	≤48 h (*n* = 26)	> 48 h (*n* = 147)	
Maternal age (years)	33.0 ± 4.0	31.3 ± 4.0	0.047
Nulliparity	35% (9)	66% (97)	0.003
Gestational age at sampling (weeks)	30.7 ± 2.6	30.0 ± 2.7	0.195
Gestational age at delivery (weeks)	30.7 ± 2.5	36.1 ± 4.0	< 0.001
Serum C-reactive protein (mg/L)	14.3 ± 15.2	8.2 ± 13.4	0.003
Cervical length on ultrasonography (mm)	13.8 ± 10.9	26.7 ± 11.1	< 0.001
Plasma C3a (μg/mL)	12.9 ± 7.9	12.9 ± 7.7	0.863
Plasma C5a (ng/mL)	41.0 ± 14.0	45.6 ± 12.5	0.237
Plasma interleukin-6 (pg/mL)	11.7 ± 12.0	4.0 ± 4.2	< 0.001
Plasma matrix metalloproteinase-9 (ng/mL)	251.0 ± 253.9	207.6 ± 175.9	0.858
Use of tocolytic agents	89% (23)	95% (139)	0.242
Use of antibiotics	27% (7)	31% (45)	0.706
Use of antenatal corticosteroids	89% (23)	76% (112)	0.165
Clinical chorioamnionitis	3.8% (1)	0% (0)	0.017
Histological chorioamnionitis^a^	50% (13/26)	35% (26/75)	0.169
Positive amniotic fluid cultures	19% (5)	7.5% (11)	0.057
Amniotic fluid interleukin-6 (ng/mL)	11.7 ± 21.4	2.8 ± 8.9	< 0.001

Data are given as mean ± standard deviation or % (n/N)

^a^Data for the histologic evaluation of the placenta were only available for 101 of the 173 women because delivery took place at another institution in 5 cases, and histologic evaluation of the placenta was not performed in 67 cases because of our institutional policy that only the placentas from preterm deliveries are sent for histopathologic examination

Multiple logistic regression analyses were performed to further examine the relationship between the various proteins in plasma, intra-amniotic infection, and imminent preterm delivery after adjusting for the effects of baseline variables. Regarding the prediction of intra-amniotic infection, the following variables were entered into the multivariate logistic regression analysis as significant predictors in the univariate analyses (*P* < 0.1): gestational age at sampling, serum CRP and plasma IL-6 levels, and cervical length. In this model, all continuous predictors were entered as dichotomous variables using the cut-off values derived from the ROC curves. The optimal cutoff values for gestational age at sampling, serum CRP level, plasma IL-6 level, and cervical length were ≤ 29.0 weeks, ≥11.2 mg/L, ≥4.8 pg/mL, and ≤ 20.6 mm, respectively. A multivariate logistic regression model indicated that only a high plasma IL-6 level was independently associated with intra-amniotic infection (Table 3). Likewise, when imminent preterm delivery was used as an outcome measure, the following dichotomized variables were used for logistic regression analysis: older age (≥33 years); multiparity, high level of serum CRP (≥3.5 mg/L), high level of plasma IL-6 (≥4.8 pg/mL), and short cervical length (≤20.0 mm). Logistic regression showed that a high plasma IL-6 level was significantly associated with imminent preterm delivery, even after adjustment for other confounders, including high serum CRP levels, short cervical length, and multiparity (Table 3).

**Table 3:** Multivariate logistic regression of potential predictors of intra-amniotic infection and imminent spontaneous preterm delivery^a^

Predictors	Odds ratios	95% Confidence interval	*P-*value
Intra-amniotic infection			
Early gestational age at sampling (≤ 29.0 weeks)	3.212	0.909–11.354	0.070
Very high serum CRP level (≥ 11.2 mg/L)	3.459	0.937–12.770	0.063
High plasma interleukin-6 level (≥ 4.8 pg/mL)	4.573	1.184–17.661	0.027
Short cervical length (≤ 20.6 mm)	2.492	0.733–8.472	0.144
Imminent preterm delivery			
Older age (≥ 33 years)	1.841	0.593–5.716	0.291
Multiparity	3.888	1.242–12.175	0.020
High serum CRP level (≥ 3.5 mg/L)	5.275	1.145–24.299	0.033
High plasma interleukin-6 level (≥ 4.8 pg/mL)	6.515	2.114–20.078	0.001
Short cervical length (≤ 20.0 mm)	8.334	2.591–26.812	< 0.001

*CRP* C-reactive protein

^a^All continuous predictors were entered as dichotomous variables using the cut-off values derived from the receiver-operating characteristic curves to predict each outcome

**Fig. 1:**
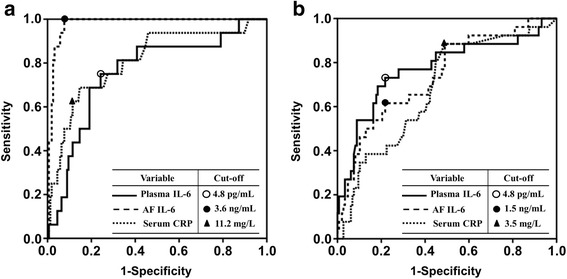
Receiver-operating characteristic curves in predicting (**a**) intra-amniotic infection and (**b**) imminent preterm delivery. [(**a**): *P* < 0.001 between plasma IL-6 and AF IL-6; *P* = 0.414 between plasma IL-6 and CRP; (**b**): no differences (*P* > 0.1) between plasma IL-6, AF IL-6, and CRP]. IL, interleukin; AF, amniotic fluid; CRP, C-reactive protein

Fig. 1 displays the ROC curves for the plasma IL-6, AF IL-6, and serum CRP levels in predicting intra-amniotic infection (Fig. 1a) and imminent preterm delivery (Fig. 1b). In predicting intra-amniotic infection, the AUC for plasma IL-6 was significantly lower than that for AF IL-6 (*P* < 0.001), but was not different from the AUC for serum CRP (*P* = 0.414) (Fig. 1a). Differences in the AUCs between plasma IL-6, AF IL-6, and serum CRP tests were not statistically significant in predicting imminent preterm delivery (Fig. 1b).

**Fig. 2:**
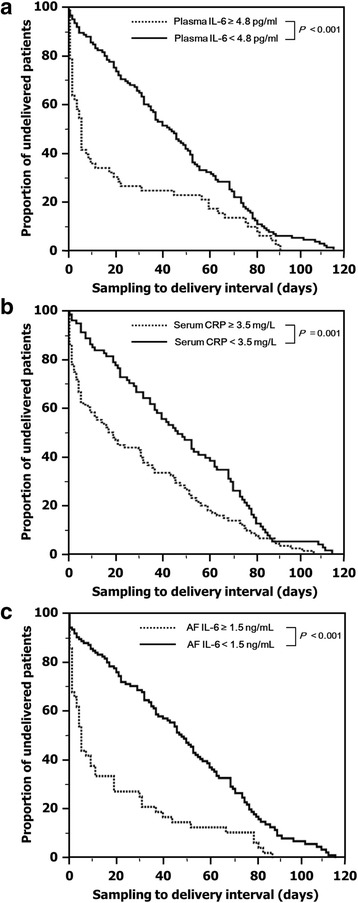
Kaplan-Meier survival estimates of the sampling-to-delivery interval for (**a**) plasma IL-6 of ≥4.8 or < 4.8 pg/mL (median, 5.00 days [95% CI, 0.70–9.30] vs. 45.00 days [95% CI, 35.31–54.69]; *P* < 0.001), (**b**) serum CRP of ≥3.5 or < 3.5 mg/L (median, 19.00 days [95% CI, 5.39–32.62] vs. 50.00 days [95% CI, 39.83–60.17]; *P* = 0.001), and (**c**) AF IL-6 of ≥1.5 or < 1.5 ng/mL (median, 5.00 days [95% CI, 1.99–8.01] vs. 47.00 days [95% CI, 38.86–55.14]; *P* < 0.001). IL, interleukin; CI, confidence interval; CRP, C-reactive protein; AF, amniotic fluid

Figure 2 shows the Kaplan–Meier estimates of the sampling-to-delivery interval for a plasma IL-6 level of ≥4.8 or < 4.8 pg/mL (Fig. 2a), serum CRP level of ≥3.5 or < 3.5 mg/L (Fig. 2b), and AF IL-6 level of ≥1.5 or < 1.5 ng/mL (Fig. 2c). Comparisons made by the log rank test were statistically significant (plasma IL-6 ≥ 4.8 pg/mL, *P* < 0.001; CRP ≥3.5 mg/L, *P =* 0.001; and AF IL-6 ≥ 1.5 ng/mL, *P* < 0.001). Similarly, using Cox proportional hazards regression model, high level of plasma IL-6 was found to be significantly associated with the sampling-to-delivery interval after being controlled for multiparity, high level of serum CRP, short cervical length, and antenatal corticosteroids (hazard ratio: 2.04; 95% confidence interval, 1.36–3.05, *P* = 0.001).

Table 4 shows the diagnostic values of plasma IL-6, AF IL-6, serum CRP, and cervical length for predicting intra-amniotic infection and imminent preterm delivery. Plasma IL-6 was less sensitive and specific than AF IL-6 (McNemar’s test, *P* < 0.001), less specific than CRP (*P* = 0.005), and equivalent in sensitivity to CRP (*P* = 0.687) for detecting intra-amniotic infection. For detection of imminent preterm delivery, the sensitivity of plasma IL-6 was not significantly different from that of AF IL-6 (*P* = 0.508) or CRP (*P* = 0.289), whereas the specificity of plasma IL-6 was significantly better than that of CRP (*P* < 0.001) but not significantly different from that of AF IL-6 (*P* = 1.000).

**Table 4:** Diagnostic indices of various factors to predict intra-amniotic infection and imminent preterm delivery

Variables	Area (±SE) under the ROC curve	95% CI	Cut-off value^a^	Sensitivity^b^ (95% CI)	Specificity^b^ (95% CI)	PPV	NPV
Intra-amniotic infection							
Plasma IL-6 (pg/mL)	0.76 ± 0.07	0.63–0.89	4.8	75.0 (50.1–93.2)	75.6 (47.6–92.7)	24.0	96.8
AF IL-6 (ng/mL)	0.98 ± 0.01^c^	0.96–1.00	3.6	100 (79.4–100.0)^d^	92.4 (87.0–96.0)^d^	57.1	100
Serum CRP (mg/L)	0.81 ± 0.06	0.69–0.93	11.2	62.5 (35.4–84.8)	86.0 (80.0–91.0)^e^	31.3	95.7
Cervical length (mm)	0.66 ± 0.06	0.55–0.77	20.6	62.5 (35.4–84.8)	65.0 (57.0–72.4)^f^	15.4	94.4
Imminent preterm delivery							
Plasma IL-6 (pg/mL)	0.77 ± 0.06	0.66–0.88	4.8	73.1 (52.2–88.4)	78.2 (70.7–84.6)	37.3	94.3
AF IL-6 (ng/mL)	0.74 ± 0.05	0.64–0.85	1.5	61.5 (40.6–79.8)	78.2 (70.7–84.6)	33.3	92.0
Serum CRP (mg/L)	0.68 ± 0.06	0.57–0.79	3.5	88.5 (69.9–97.6)	51.0 (42.7–59.4)^d^	24.2	96.2
Cervical length (mm)	0.81 ± 0.05	0.72–0.90	20.0	76.9 (56.4–91.0)	71.4 (63.4–78.6)	32.3	94.6

*SE* standard error; *ROC* receiver-operating characteristic, *CI* confidence interval, *PPV* positive predictive value, *NPV* negative predictive value; *IL-6* interleukin-6, *AF* amniotic fluid, *CRP* C-reactive protein

^a^Cut-off values corresponding to the highest sum of sensitivity and specificity

^b^Values are given as % (95% CI)

^c^*P* < 0.001 compared to plasma IL-6 by the method of DeLong et al.

^d^*P* < 0.001 compared to plasma IL-6 by McNemar’s test

^e^*P* < 0.01 compared to plasma IL-6 by McNemar’s test

^f^*P* < 0.05 compared to plasma IL-6 by McNemar’s test

## Discussion

Our principal findings are as follows: (i) in women with preterm labor, plasma IL-6 is moderately predictive of intra-amniotic infection and imminent preterm delivery; (ii) plasma IL-6 has a worse overall diagnostic performance than that of AF IL-6 and a similar diagnostic performance to that of CRP (a prototype of inflammatory response) in predicting intra-amniotic infection; (iii) for prediction of imminent preterm delivery, the diagnostic performance of plasma IL-6, AF IL-6, and serum CRP was similar; and (iv) the plasma levels of MMP-9, C3a, and C5a could not predict intra-amniotic infection or imminent preterm delivery. Similar results regarding the relationship between plasma pro-inflammatory cytokines levels, intra-amniotic infection, and histologic chorioamnionitis were also documented in women with preterm labor [13, 19, 25] and pPROM [13, 16].

We found that an elevated plasma level of IL-6 predicted intra-amniotic infection in women with preterm labor. Our findings confirm those previously reported by Cobo et al., demonstrating that women with preterm labor (22 + 0 to 31 + 6 weeks of gestation) and intra-amniotic infection had higher levels of maternal serum IL-6 than did those without intra-amniotic infection [13]. Moreover, we showed that the association between plasma IL-6 and intra-amniotic infection remained unchanged in multivariate logistic analysis after adjusting for gestational age at sampling and CRP. Moreover, to the best of our knowledge, our study is the first to compare plasma IL-6 with AF IL-6 and serum CRP in terms of overall accuracy in identifying intra-amniotic infection. We found that plasma IL-6 has a worse overall diagnostic performance than that of AF IL-6 in predicting intra-amniotic infection. These findings suggest that microbial invasion of the amniotic cavity (MIAC) in women with preterm labor elicits a much weaker inflammatory response (mediated by IL-6) in the maternal plasma compartment than in the AF compartment and, thus, the inflammatory biomarkers in the maternal plasma cannot precisely reflect the infectious status of the amniotic cavity. In fact, these observations are natural because microorganisms in the amniotic cavity can directly increase the production of the chemical mediators in the original site (i.e., AF), where the host immune response to microorganisms occurs. Accordingly, to determine maternal plasma biomarkers, future studies based on histologic chorioamnionitis rather than MIAC are warranted because acute inflammatory processes of the placenta are of maternal origin [26] and may be reflected in the plasma.

As previously reported [17, 19, 27, 28], we found that elevated plasma IL-6 levels predict imminent preterm delivery with a diagnostic performance similar to that of AF IL-6. Further, multivariate logistic regression analysis indicated that factors such as multiparity, high serum CRP level, and short cervical length, as well as high plasma IL-6 levels, were also independently associated with an increased risk of imminent preterm delivery, similar to the findings of Tsiartas et al. [29]. Taken together, these findings suggested that the etiology of preterm labor leading to SPTD may be multifactorial. Thus, a combination of factors contributing to the pathophysiology of preterm birth may enhance the ability to predict SPTD. We believe that, unlike predicting intra-amniotic infection, a highly sensitive and specific test for SPTD can be created by developing novel biomarkers in maternal plasma, alone or in combination with clinical parameters, thus allowing targeted interventions.

Importantly, we found that serum CRP (the most used infection/inflammation marker) has a predictive power similar to that of plasma IL-6 for predicting intra-amniotic infection and is similar to that of plasma IL-6 and AF IL-6 for predicting imminent preterm delivery. Similarly, our previous study found that serum CRP has a diagnostic performance similar to that of IL-6 and MMP-9 in AF for the detection of histologic chorioamnionitis [7]. These may be important from a clinical perspective, as the measurement of serum CRP is simple, noninvasive, and inexpensive and may provide information to improve maternal and infant health and patient counseling. However, it is not a sensitive or specific marker for either intra-amniotic infection or SPTD [30, 31].

MMP-9 is an important zinc-dependent protease in the MMP superfamily, which is involved in inflammation, cytokine processing, and tissue remodeling [32]. Therefore, several studies have investigated the high expression of MMP-9 in AF associated with intra-amniotic infection, and found AF MMP-9 levels to be the strongest predictor of intra-amniotic infection in preterm labor [7, 8]. However, to our knowledge, no studies have investigated the changes in MMP-9 levels in maternal plasma in relation to intra-amniotic infection and imminent preterm delivery in the setting of preterm labor. We observed that intra-amniotic infection was not associated with a change in plasma MMP-9 levels. Moreover, regarding imminent preterm delivery, no significant change in MMP-9 levels was observed, consistent with the findings of a previous study conducted in an asymptomatic cohort [27].

The complement system is pivotal for innate immunity, the first line of defense against invading pathogens [33]. Thus, the relationship between complement activation, preterm birth, and intra-uterine infection/inflammation has been investigated. Interestingly, Lynch et al. found that elevated plasma levels of the complement activation fragment C3a and Bb in early gestation (< 20 weeks) are independent predictive factors for SPTD [34, 35]. Soto et al. reported that women with preterm labor and intra-amniotic infection have higher median maternal plasma C3a and C5a levels than those with preterm labor without intra-amniotic infection, whereas there was no difference in the plasma C3a and C5a levels between women with preterm labor who delivered at term and those with preterm delivery (< 37 weeks) [18]. However, our study did not reveal any association between plasma complement C3a/C5a and intra-amniotic infection or imminent preterm delivery, suggesting that the maternal immune system in the plasma compartment related to complement does not respond to the microbes or microbial products in the amniotic cavity and is not implicated in the process of preterm parturition. This discrepancy may be attributed to differences in the populations studied (asymptomatic women versus those presenting with symptoms of preterm labor), study design (cross-sectional versus longitudinal), and sample size.

Our study has several limitations. First, it was a retrospective study conducted at a single center, limiting the generalizability of our findings. Second, we did not fully characterize the inflammatory and immune proteins in plasma but only measured the relevant proteins that had the most potential as a plasma biomarker of high accuracy to detect intra-amniotic infection and preterm birth [13, 17, 18, 29, 34]. Third, in line with our previous studies [6, 20], only *Ureaplasma urealyticum* and *Mycoplasma hominis* were detected in the AF, although the reason is unclear. However, considering the fact that various aerobic and anaerobic bacteria were recovered from the AF of women with pPROM in our hospital during nearly the same period [36, 37], we believe that isolation of only mycoplasma in the current study is not associated with the technical problems of aerobic and anaerobic cultures in our hospital. Fourth, the treatment regimen for management of preterm labor varied according to the study period and the obstetrician, which may lead to affecting the outcome of interest and statistical analyses.

## Conclusions

In conclusion, maternal plasma IL-6 independently predicts intra-amniotic infection in women with preterm labor; however, it has worse diagnostic performance than that of AF IL-6 and similar performance to that of serum CRP. For prediction of imminent preterm delivery, the diagnostic performance of plasma IL-6, AF IL-6, and serum CRP were similar. Plasma MMP-9, C3a, and C5a levels did not predict intra-amniotic infection or imminent preterm delivery. Therefore, the assessment of inflammatory and immune markers in the maternal plasma may have limited clinical usefulness for identifying intra-amniotic infection in women with preterm labor. Further studies should utilize cervicovaginal fluid, rather than maternal plasma, to help identify new non-invasive biomarkers for intra-amniotic infection.

## Abbreviations

AF: Amniotic Fluid
AUC: Area Under the Curve
CI: Confidence Interval
CRP: C-Reactive Protein
ELISA: Enzyme-Linked Immunosorbent Assay
IL: Interleukin
MIAC: Microbial Invasion of the Amniotic cavity
MMP: Matrix Metalloproteinase
NPV: Negative Predictive Value
pPROM: Premature Rupture of the Membranes
PPV: Positive Predictive Value
ROC: Receiver-Operating Characteristic
SE: Standard Error
SPTD: Spontaneous Preterm Delivery
